# Cardio-haemodynamic assessment and venous lactate in severe dengue: Relationship with recurrent shock and respiratory distress

**DOI:** 10.1371/journal.pntd.0005740

**Published:** 2017-07-10

**Authors:** Sophie Yacoub, Trieu Huynh Trung, Phung Khanh Lam, Vuong Huynh Ngoc Thien, Duong Ha Thi Hai, Tu Qui Phan, Oanh Pham Kieu Nguyet, Nguyen Than Ha Quyen, Cameron Paul Simmons, Christopher Broyd, Gavin Robert Screaton, Bridget Wills

**Affiliations:** 1 Oxford University Clinical Research Unit, Wellcome Trust Major Overseas Programme, Ho Chi Minh City, Vietnam; 2 Department of Medicine, Imperial College London, London, United Kingdom; 3 Intensive Care Unit, Hospital for Tropical Diseases, Ho Chi Minh City, Vietnam; 4 Department of Microbiology and Immunology, University of Melbourne, Melbourne, Australia; 5 Faculty of Medicine, National Heart and Lung Institute, Imperial College London, London, United Kingdom; 6 Nuffield Department of Medicine, University of Oxford, Oxford, United Kingdom; University of Rhode Island, UNITED STATES

## Abstract

**Background:**

Dengue can cause plasma leakage that may lead to dengue shock syndrome (DSS). In approximately 30% of DSS cases, recurrent episodes of shock occur. These patients have a higher risk of fluid overload, respiratory distress and poor outcomes. We investigated the association of echocardiographically-derived cardiac function and intravascular volume parameters plus lactate levels, with the outcomes of recurrent shock and respiratory distress in severe dengue.

**Methods/Principle findings:**

We performed a prospective observational study in Paediatric and adult ICU, at the Hospital for Tropical Diseases (HTD), Ho Chi Minh City, Vietnam. Patients with dengue were enrolled within 12 hours of admission to paediatric or adult ICU. A haemodynamic assessment and portable echocardiograms were carried out daily for 5 days from enrolment and all interventions recorded.

102 patients were enrolled; 22 patients did not develop DSS, 48 had a single episode of shock and 32 had recurrent shock. Patients with recurrent shock had a higher enrolment pulse than those with 1 episode or no shock (median: 114 vs. 100 vs. 100 b/min, P = 0.002), significantly lower Stroke Volume Index (SVI), (median: 21.6 vs. 22.8 vs. 26.8mls/m^2^, P<0.001) and higher lactate levels (4.2 vs. 2.9 vs. 2.2 mmol/l, P = 0.001). Higher SVI and worse left ventricular function (higher Left Myocardial Performance Index) on study days 3–5 was associated with the secondary endpoint of respiratory distress. There was an association between the total IV fluid administered during the ICU admission and respiratory distress (OR: 1.03, 95% CI 1.01–1.06, P = 0.001). Admission lactate levels predicted patients who subsequently developed recurrent shock (P = 0.004), and correlated positively with the total IV fluid volume received (rho: 0.323, P = 0.001) and also with admission ALT (rho: 0.764, P<0.001) and AST (rho: 0.773, P<0.001).

**Conclusions/Significance:**

Echo-derived intravascular volume assessment and venous lactate levels can help identify dengue patients at high risk of recurrent shock and respiratory distress in ICU. These findings may serve to, not only assist in the management of DSS patients, but also these haemodynamic endpoints could be used in future dengue fluid intervention trials.

## Author summary

Dengue is a viral illness that can lead to severe and potentially fatal complications. The most common complication is fluid leakage from blood vessels, which can cause low blood pressure or dengue shock syndrome (DSS). The majority of patients recover with simple intravenous fluid replacement, however in approximately 30% of DSS cases, recurrent episodes of shock occur, and these patients have a higher risk of fluid overload, respiratory distress and death. We investigated whether using portable echocardiograms (Echo) in the intensive care unit (ICU) to assess cardiac function and intravascular volume parameters as well as blood lactate levels, can help identify these patients. We found patients who developed recurrent shock had higher heart rates and lower Stroke Volume Index (SVI), and higher lactate levels at enrolment than those with 1 episode or no shock. Higher SVI and worse cardiac function after 3 days in ICU was associated with respiratory distress. Admission lactate levels predicted patients who subsequently developed recurrent shock and correlated positively with the total IV fluid volume received. These results demonstrate that Echo-derived intravascular volume assessment and venous lactate levels can help identify dengue patients at high risk of poor outcomes in the ICU, and could assist in the management of severe dengue.

## Introduction

Dengue is a *flaviviral* infection that causes substantial morbidity in endemic areas, with 96 million clinically apparent cases each year [1]. Although the majority of infections result in a self-limiting febrile illness, 1–5% of cases can experience more severe manifestations, in the form of organ impairment, coagulopathy and plasma leakage which may lead to intravascular volume depletion and dengue shock syndrome (DSS). The plasma leakage usually resolves around defervescence, and the extravasated fluid then gets reabsorbed, when fluid overload in the form of massive pleural effusions or pulmonary oedema can occur. The resulting respiratory compromise has been associated with an increased risk of death in adult [2] and paediatric severe dengue [3]. The current treatment of DSS is supportive, with careful intravenous fluid replacement. The majority of patients recover after a single crystalloid bolus and in experienced centres the mortality rate is less than 1% [4]. However, in approximately 30% of DSS cases, recurrent episodes of shock occur, which require more intensive treatment with larger volumes of intravenous fluids including colloid boluses; these patients have a higher risk of fluid overload, respiratory distress and poor outcomes [5]. Identifying such individuals early and investigating other potential contributing factors for recurrent shock is needed. Although the main mechanism of DSS is hypovolaemia, it is becoming increasingly recognized that myocardial impairment may play a role in the haemodynamic instability and potentially could contribute to recurrent shock [6, 7]. Cardiac manifestations of dengue are diverse and include functional myocardial impairment, arrhythmias and myocarditis, however the clinical significance of these in DSS has not been well studied [8].

We have shown previously that systemic microvascular dysfunction occurs in more severe dengue infections, but the effect on end-organ perfusion has not been evaluated [9]. Lactate levels can be representative of tissue perfusion and elevated levels are associated with organ failure and predict mortality in septic shock [10, 11]. Serum lactate levels and their prognostic significance in dengue shock syndrome have only been evaluated in small studies and in adults [12–14].

In this study we investigated the association of echocardiographically-derived cardiac function and intravascular volume parameters as well as lactate levels with the clinical outcomes of recurrent shock and respiratory distress in adults and children admitted to ICU with dengue. We hypothesised that DSS patients with cardiac dysfunction and elevated lactate levels would be more likely to develop recurrent shock and also to experience iatrogenic fluid overload and respiratory distress.

## Methods

This prospective observational study was performed at the Hospital for Tropical Diseases (HTD), Ho Chi Minh City, Vietnam. Individuals aged above 3 years were screened for enrolment if they were admitted to either paediatric or adult Intensive Care Unit (ICU) with a clinical diagnosis of dengue with warning signs or severe dengue [15], and were within 12 hours from ICU admission. In Vietnam patients aged 15 or more are admitted to adult ICU. All patients were reviewed daily until hospital discharge or for up to 5 days from enrolment; at each assessment standardized clinical information was recorded including clinical symptoms and signs, vital signs and all interventions. The amount and type of all intravenous fluids were documented. Portable echocardiograms were performed as soon as feasible after enrolment, and then daily until discharge from ICU. The patients were followed up 10–14 days later.

### Ethics statement

Ethical approvals were obtained from the Oxford Tropical Research Ethics Committee and the Ethics Review Committee at HTD, and written informed consent was obtained from all participants or the parent/guardian of children.

### Laboratory parameters

A full blood count was performed daily and at follow-up. A biochemistry sample for liver and renal function was performed at enrolment and subsequently depending on clinical need. An un-cuffed venous blood sample was taken at enrolment for venous lactate which was processed within 30 minutes of collection.

Dengue diagnostics: Commercial IgM and IgG serology assays (Capture ELISA, Panbio, Australia) were performed on batched acute and convalescent plasma. In addition RT-PCR was performed on the enrolment sample to identify the DENV serotype and measure plasma viraemia levels [16]. Patients were defined as having dengue if the RT-PCR was positive or if the IgM assays were positive at enrolment, or IgM seroconversion between paired specimens and on the basis of their clinical picture. Patients with negative tests at enrolment, but for whom convalescent plasma was not available, were considered unclassifiable.

### Portable echocardiograms

Echocardiograms were performed at the bedside by one of the investigators (SY, HT, VN), using an M-turbo system (FUJIFILM SonoSite, Inc, USA) with cardiac settings. The Echocardiograms were performed daily and at follow-up 14 days later. The exam included two-dimensional, M-mode and Doppler studies. More detailed methodology can be found elsewhere [6]. All images were stored digitally and a selection reviewed by a cardiologist (CB) in the United Kingdom. The inter- and intra- user variability was checked at regular intervals and was consistently <10%.

### Statistical analysis and endpoint definitions

#### Endpoints

The primary endpoint was recurrent shock, which was defined as occurrence of more than one episode of clinical shock during the ICU admission. Shock episodes were recorded by the study doctors each day using the following definition; hypotension for age or pulse pressure of 20 mmHg or less with signs of circulatory compromise, requiring additional fluid bolus administration or refractory shock, requiring additional inotropic support. The secondary endpoint was respiratory distress, defined as the need for oxygen therapy or respiratory support. Cardiac dysfunction was defined as an ejection fraction <55% and/or left/right myocardial performance index (L/RMPI >0.5).

### Statistical analysis

Linear regression models were used for the initial analysis, with each cardio-haemodynamic parameter as the outcome and shock status as covariate. The analysis was adjusted for age, gender, and day of illness at ICU admission. Logistic regression was used for the prognostic models, predefined candidate variables at enrolment were used to predict recurrent shock/respiratory distress. Associations between the parameters were assessed by partial correlations controlling for the following potential confounding variables: age, sex and day of illness at enrolment, study day of measurement. Significance of partial correlations was assessed based on their Fisher transformation and corresponding bootstrap standard errors. The cluster bootstrap which resamples patients rather than samples accounted for multiple measurements per patient. To informally adjust for multiplicity, a significance level of 0.01 was used for all comparisons. All analyses were performed with the statistical software R version 3.2.2 and the companion package geepack version 1.2–0.

## Results

One hundred and three patients were enrolled between September 2014 and September 2015, 1 patient was excluded from the analysis due to an inconclusive diagnosis and 88 had serial echocardiograms (S1 Fig). The median age was 11 years (IQR 8–14 years). The median time from ICU admission to enrolment was 0.1 hours (IQR: 0.0–1.0 hour), and from enrolment to first echo was 2.5 hours (IQR 1.0–9.3 hours). Twenty-two patients did not develop DSS, 80 patients had DSS, of which 48 had a single episode and 32 had recurrent episodes of shock (4 of these 32 patients required inotropes for refractory shock). Of the patients with 1 episode of shock 34/48 had shock at ICU admission and 14/48 developed shock; median time of 1.75 hours (IQR: 0.75–4.08 hours) after admission. The patients with recurrent shock, 27/32 had shock at ICU admission and 5/32 developed their first episode of shock; median of 1.92 hours, (0.06–6.88 hrs) after admission. The patients who were not in shock at ICU admission, were admitted for more intensive monitoring and/or fluid therapy due to the presence of several warning signs. Nineteen patients developed the secondary endpoint of respiratory distress, 10 (53%) on day 2, 8 (42%) on day 3 and 1 (5%) on day 4, all had radiological evidence of pleural effusions but not pulmonary oedma (S1A Fig). Seven patients had major bleeding, all following shock, 6/7 with recurrent shock and 1/7 with 1 episode of shock. Of the 97 patients for whom RT-PCR was performed, 65 were positive with the following serotypes: 52 DENV-1 (80%); 10 DENV-2 (15%; and 3 DENV-4 (5%). There was 1 death in the study population in adult ICU. 63/102 (62%) were admitted to ICU straight from clinic/community, 39/102 were admitted from the general ward, after a median of 22 hours, but no intravenous fluids were administered on the general wards.

At enrolment, platelet counts and albumin levels were significantly lower, and AST levels significantly higher, in patients who later experienced recurrent shock than those who did not. Lactate levels were also significantly higher in patients who went on to have recurrent episodes of shock than those who had 1 episode or no shock (Table 1). Hyperlactatemia, using a cut-off of >4 mmol/l, was present in 17/32 (53.1%) of the individuals with recurrent shock, 8/48 (16.7%) of the patients with 1 shock episode and was absent in all patients without shock. Considering the 88 patients that had serial echocardiograms, 24/88 (27%) had evidence of left ventricular dysfunction and 6/8 (7%) right ventricular dysfunction. A higher proportion of adults had left and right ventricular dysfunction, 11/16 (69%) and 4/16 (25%) compared to children, 13/72 (18%) and 2/72 (3%).

**Table 1:** Enrollment characteristics for dengue patients in ICU.

	All patients (n = 102)		No shock (n = 22)		Shock (n = 48)		Recurrent shock (n = 32)		p
Characteristic	n		n		n		n		
Age (yrs)	102	11 (8, 14)	22	12 (10, 20)	48	11 (8, 14)	32	10 (8, 13)	0.583
Female	102	48 (47.1%)	22	11 (50.0%)	48	22 (45.8%)	32	15 (46.9%)	0.966
Illness day	102	6 (5, 6)	22	6 (5, 6)	48	6 (5, 6)	32	6 (5, 6)	0.237
Platelets (10^9^/L)	99	29(18, 41)	22	37 (30, 62)	46	28 (19, 40)	31	27 (15, 33)	0.011
White cells (10^9^/L)	99	4.6 (3.0, 5.8)	22	4.9 (3.4, 5.8)	46	4.6 (3.0, 6.3)	31	3.4 (3.0, 5.6)	0.695
HCT (%)	98	44.6 (41.6,48.6)	22	42.0 (40.7, 45.6)	45	44.7 (42.5, 48.8)	31	46.6 (41.5, 50.0)	0.076
Albumin(g/L)	98	33.5 (29.2, 37.3)	22	38.0 (33.3, 41.0)	46	33.5 (29.4, 37.0)	30	30.1 (23.6,34.8)	<0.001
AST(U/L)	92	164 (104, 343)	20	102 (80, 162)	45	217 (106, 384)	27	219 (129, 540)	0.001
ALT(U/L)	92	86 (41–161)	20	63 (32–96)	45	98 (46–160)	27	112 (45–266)	0.041
Lactate (mmol/l)	98	2.9 (2.2, 3.9)	22	2.2 (2.0, 2.7)	46	2.9 (2.2, 3.6)	30	4.2 (2.6, 4.7)	<0.001
Fluid accumulation	102	64 (62.7%)	22	5 (22.7%)	48	31 (64.6%)	32	28 (87.5%)	<0.001
**Outcomes**									
Total IV Fluid	84	3155 (1845, 5211)	5	0 (0, 0)	47	3020 (2198, 4337)	32	5876 (4257, 7300)	<0.001
Colloid bolus	102	30 (35.7%)	22	0 (0.0%)	47	5 (10.6%)	32	25 (78.1%)	<0.001
>1 Colloid bolus	102	19 (18.6%)	22	0 (0.0%)	48	0 (0.0%)	32	19 (59.4%)	
Respiratory Distress	102	19 (18.6%)	22	0 (0.0%)	48	4 (8.3%)	32	15 (46.9%)	<0.001
Inotropes	102	4 (3.9%)	22	0 (0.0%)	48	0 (0.0%)	32	4 (12.5%)	0.010

Data is presented as absolute count (%) for categorical variables and median (IQR) for continuous data. P-values are based on Kruskal-Wallis (continuous data) and Fisher's exact test (categorical data).

Fluid accumulation: Clinical pleural effusion and/or ascites

### Enrollment cardio-haemodynamic parameters

Patients who developed recurrent shock had a higher enrolment pulse than those with 1 episode of shock or no shock (median: 114 vs. 100 vs. 100 b/min, P = 0.002), and reduced pulse pressure (PP) (median: 20 vs. 20 vs. 30 mmHg, P = 0.001) (Table 2). There was a significantly lower Stroke Volume Index (SVI) in the patients with recurrent shock versus patients with and without 1 shock (median: 21.6 vs. 22.8 vs. 26.8ml/m^2^, P = 0.002).

**Table 2:** Enrollment cardiac function and Haemodynamics in dengue patients on admission to the Intensive Care Unit.

n		All patients (N = 102) n		No shock (N = 22) n		Shock (N = 48) n		Recurrent shock (N = 32)	p
Pulse (b/min)	102	101 (92, 120)	22	100 (80, 106)	48	100 (92, 114)	32	114 (100, 130)	0.003
MAP (mmHg)	102	83 (77, 87)	22	73 (72, 80)	48	80 (73, 88)	32	80 (70, 86)	0.506
PP (mmHg)	102	20 (20, 30)	22	30 (30, 40)	48	20 (20, 30)	32	20 (15, 20)	<0.001
SVI (ml/m^2^)	68	22.6 (20.1, 25.3)	14	26.8 (23.2, 28.4)	32	22.8 (20.6, 25.3)	22	21.6 (19.4, 23.0)	0.002
CI (L/min/m^2^)	68	2.2 (1.9, 2.5)	14	2.3 (2.2, 2.7)	32	2.0 (1.8, 2.3)	22	2.2 (2.0, 2.5)	0.035
IVCCI	64	0.39 (0.34, 0.48)	13	0.37 (0.31, 0.40)	32	0.40 (0.35, 0.49)	19	0.40 (0.35, 0.47)	0.323
LVDd (cm)	68	3.7 (3.3, 4.0)	14	3.6 (3.3, 4.2)	32	3.7 (3.3, 4.0)	22	3.5 (3.3, 3.9)	0.914
EF (%)	68	66.3 (61.9, 69.0)	14	66.8 (61.7, 69.0)	32	66.7 (62.9, 69.6)	22	65.8 (60.7, 68.1)	0.711
LMPI	68	0.36 (0.27, 0.48)	14	0.36 (0.27, 0.50)	32	0.41 (0.26, 0.48)	22	0.34 (0.28, 0.41)	0.942
RMPI	66	0.18 (0.12, 0.26)	14	0.14 (0.12, 0.21)	32	0.18 (0.12, 027)	20	0.18 (0.12, 0.26)	0.278

Data is presented as absolute count (%) for categorical variables and median (IQR) for continuous data. P-values are based on Kruskal-Wallis (continuous data) and Fisher's exact test (categorical data).

### Association of cardio-haemodynamic parameters with shock status over ICU admission

**Fig 1:**
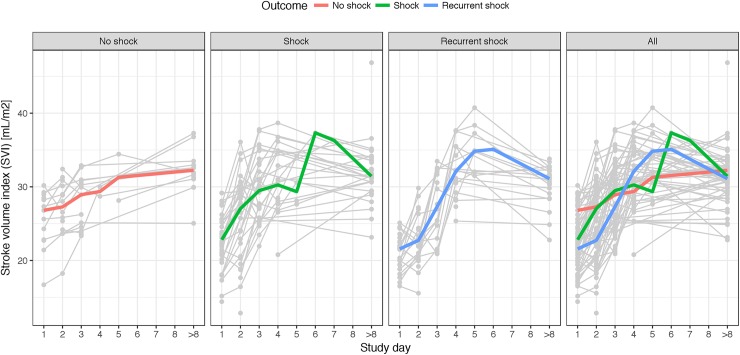
“Stroke Volume Index over ICU admission in patients with no shock, shock and recurrent shock.” Graph showing the dynamics of SVI by study day for 87 patients. Grey lines are changes of SVI for each patient. Colored lines connect medians of SVI on each study day for each patient group.

SVI was significantly lower at enrolment (study day 1) for patients with recurrent shock compared with no shock (median: 21.6 vs. 26.8mls/m^2^, P<0.001) and also between patients with shock compared with no shock (median: 22.8 vs. 26.8mls/m^2^, P = 0.001) (Table 3, Fig 1).

**Table 3:** Cardio-haemodynamic variables in patients with and without shock and recurrent shock, over ICU admission.

		No shock		Shock		Recurrent shock		Recurrent shock vs. shock			Recurrent shock vs. no shock	
	n		n		n		Effect	95% CI	p	Effect	95% CI	p
**SVI ml/m**^**2**^												
Day 1	14	26.8 (23.2, 28.4)	32	22.8 (20.6, 25.3)	22	21.6 (19.4, 23.0)	-1.25	(-3.09, 0.60)	**0.185**	-4.63	(-6.89, -2.36)	**<0.001**
Day 2	16	27.2 (24.1, 29.3)	34	27.1 (21.0, 29.4)	22	22.8 (20.6, 24.3)	-3.00	(-5.50, -0.50)	**0.019**	-4.34	(-7.30, -1.38)	**0.004**
Day 3	15	28.9 (24.2, 30.7)	34	29.5 (26.1, 32.8)	16	27.0 (23.0, 31.7)	-2.14	(-4.67, 0.39)	**0.098**	0.06	(-2.89, 3.02)	**0.967**
Day 4	2	29.4 (29.0, 29.7)	17	30.3 (27.7, 34.8)	17	32.1 (29.2, 35.4)	1.84	(-1.11, 4.79)	**0.221**	3.17	(-3.10, 9.43)	**0.322**
Day 5	2	31.2 (29.7, 32.8)	4	29.4 (28.0, 31.5)	8	34.8 (31.9, 37.6)	4.20	(-0.63, 9.03)	**0.088**	-1.64	(-16.68, 13.4)	**0.831**
>Day 13	14	32.2 (31.2, 33.0)	32	31.4 (29.5, 33.5)	22	31.1 (29.3, 32.2)						
**CI l/min/m**^**2**^												
Day 1	14	2.3 (2.2, 2.7)	32	2.0 (1.8, 2.3)	22	2.2 (2.0, 2.5)	0.21	(0.00, 0.42)	**0.053**	-0.18	(-0.44, 0.08)	**0.172**
Day 2	16	2.4 (2.2, 2.5)	34	2.2 (1.9, 2.5)	22	2.2 (2.0, 2.3)	-0.01	(-0.25, 0.22)	**0.923**	-0.17	(-0.44, 0.11)	**0.236**
Day 3	15	2.4 (2.1, 2.5)	34	2.5 (2.2, 2.7)	16	2.7 (2.3, 3.0)	0.17	(-0.08, 0.41)	**0.176**	0.34	(0.06, 0.62)	**0.019**
Day 4	2	2.4 (2.3, 2.4)	17	2.6 (2.2, 2.9)	17	3.0 (2.7, 3.1)	0.42	(0.03, 0.80)	**0.037**	0.64	(-0.19, 1.46)	**0.130**
Day 5	2	2.4 (2.3, 2.5)	4	2.5 (2.2, 2.8)	8	3.0 (2.5, 3.2)	0.08	(-0.35, 0.52)	**0.699**	-1.22	(-2.56, 0.12)	**0.075**
>Day13	14	2.8 (2.6, 2.9)	31	2.8 (2.6, 2.8)	22	2.7 (2.6, 2.9)						
**LMPI**												
Day 1	14	0.36 (0.27, 0.50)	32	0.42 (0.26, 0.48)	22	0.34 (0.28, 0.41)	0.00	(-0.08, 0.07)	**0.951**	0.02	(-0.08, 0.11)	**0.742**
Day 2	14	0.37 (0.30, 0.44)	26	0.37 (0.30, 0.48)	17	0.37 (0.26, 0.51)	0.01	(-0.08, 0.09)	**0.888**	0.02	(-0.08, 0.12)	**0.752**
Day 3	14	0.28 (0.20, 0.36)	26	0.35 (0.27, 0.43)	16	0.34 (0.25, 0.45)	-0.01	(-0.09, 0.08)	**0.852**	0.08	(-0.01, 0.18)	**0.085**
Day 4	2	0.30 (0.26, 0.33)	15	0.36 (0.32, 0.46)	16	0.42 (0.31, 0.51)	0.03	(-0.05, 0.11)	**0.448**	0.09	(-0.08, 0.26)	**0.300**
Day 5	2	0.33 (0.36, 0.37)	4	0.23 (0.21, 0.28)	8	0.31 (0.29, 0.35)	0.05	(-0.02, 0.13)	**0.169**	-0.09	(-0.32, 0.15)	**0.465**
>Day13	14	0.17 (0.11, 0.23)	31	0.20 (0.18, 0.23)	22	0.21 (0.19, 0.24)						
**IVCCI**												
Day 1	13	0.37 (0.31, 0.40)	32	0.40 (0.35, 0.49)	19	0.40 (0.35, 0.47)	-0.01	(-0.07, 0.05)	**0.802**	0.05	(-0.03, 0.12)	**0.222**
Day 2	14	0.31 (0.25, 0.36)	33	0.33 (0.28, 0.36)	19	0.31 (0.30, 0.37)	0.01	(-0.04, 0.06)	**0.826**	0.03	(-0.03, 0.09)	**0.285**
Day 3	15	0.29 (0.23, 0.33)	33	0.24 (0.20, 0.30)	16	0.28 (0.25, 0.32)	0.04	(0.00, 0.09)	**0.067**	0.01	(-0.04, 0.07)	**0.601**
Day 4	2	0.28 (0.25, 0.31)	18	0.23 (0.21, 0.27)	17	0.24 (0.21, 0.26)	0.00	(-0.04, 0.04)	**0.902**	-0.04	(-0.12, 0.05)	**0.397**
Day 5	2	0.22 (0.22, 0.22)	4	0.20 (0.17, 0.23)	8	0.22 (0.21, 0.25)	0.02	(-0.03, 0.07)	**0.429**	-0.05	(-0.21, 0.10)	**0.508**
>Day13	14	0.22 (0.15, 0.23)	32	0.21 (0.18, 0.23)	22	0.20 (0.18, 0.24)						

Data is presented as median (IQR). Each row corresponds to comparison of each variable by admission day based on linear regression, with adjustment for age, sex, day of illness at enrolment. Effect (and 95%CI, p) corresponds to mean difference in variable of interest between recurrent shock and shock and also recurrent shock and no shock. Day = study day, SVI = Stroke Volume Index, CI = Cardiac Index, LMPI = Left Myocardial Performance Index, IVCCI = Inferior Vena Cava collapsibility Index.

Using linear regression there was a significant trend test for SVI between the shock groups on day 1 (P<0.001) and day2 (P = 0.003).

There was a significantly lower cardiac index (CI) between patients with shock compared to no shock on the first study day. The SVI remained lower for patients with recurrent shock versus no shock on study day 2 (median: 22.8 vs. 27.2 mls/m^2^, P = 0.004). A non-significant trend for higher CI and LMPI was observed on study day 3 in the recurrent shock patients compared to no shock.

### Association of cardio-haemodynamic parameters and respiratory distress over ICU admission

Higher SVI on study day 4 was associated with the secondary endpoint of respiratory distress, as well as a trend for higher CI and respiratory distress (S1 Table). The majority of patients only received IV fluids on days 1–2 days (S2 Table), so the higher SVI on days 4 and 5 likely represents fluid re-absorption rather than iatrogenic causes. On study days 3–5, worse left ventricular function (higher LMPI) was associated with respiratory distress. There was an association between the total IV fluid administered during the ICU admission and respiratory distress (OR: 1.03, 95% CI 1.01–1.06, P = 0.001). Respiratory distress presented early (study day 2) in half of the patients, all had evidence of bilateral pleural effusions, suggesting plasma leakage likely causes early respiratory distress in ICU which is later compounded by fluid re-absorption and myocardial impairment on study days 3–5.

### Prognostic value of cardio-haemodynamics and laboratory variables for recurrent shock and respiratory distress

Enrolment lactate levels predicted patients who subsequently developed recurrent shock compared to those who did not (Table 4). In addition, higher enrolment lactate levels were also found to predict patients developing respiratory distress (3.9 vs. 3.0 mmol/l, OR 1.46, 95% CI 1.09–2.12, P = 0.008).

**Table 4:** Enrollment cardio-haemodynamic and laboratory variables for predicting recurrent shock.

	n	One episode Shock (n = 48)	n	Recurrent Shock (n = 32)	OR	(95% CI)	p value
SVI ml/m^2^	32	22.8 (20.6, 25.3)	22	21.6 (19.4, 23.0)	0.88	(0.73, 1.05)	0.173
CI l/min/m^2^	32	2.0 (1.8, 2.3)	22	2.2 (2.0, 2.5)	6.31	(1.21, 40.97)	0.028
LMPI	32	0.41 (0.26, 0.48)	22	0.34 (0.28, 0.41)	0.77	(0.01, 56.19)	0.906
RMPI	32	0.2 (0.1, 0.3)	19	0.2 (0.1, 0.3)	0.73	(0.00, 175.70)	0.910
IVCCI	32	0.40 (0.35, 0.49)	19	0.40 (0.35, 0.47)	1.97	(0.00, 1014.93)	0.828
HCT (%)	45	44.7 (42.5, 48.8)	31	46.6 (41.5,50.0)	1.04	(0.95, 1.15)	0.405
PLT (10^9^/L)	46	28 (19, 41)	31	27 (15, 33)	0.99	(0.97,1.01)	0.534
Lactate (mmol/l)	46	2.9 (2.2, 3.6)	30	4.2 (2.6, 4.7)	1.46	(1.11, 2.07)	0.004
Log2Lactate	46	1.6 (1.1, 1.9)	30	2.1 (1.4, 2.2)	2.71	(1.29, 6.41)	0.008

Data are presented as absolute count (%) for categorical variables and median (IQR) for continuous data. All analyses were based on univariate logistic regression model, adjusted for age, sex, illness day at enrolment.

### Correlation of cardio-haemodynamic variables and lactate levels

The SVI correlated with other parameters of intravascular volume including inferior vena cava collapsibility index (IVCCI) with a negative correlation (rho -0.491, P<0.001) and left ventricular end-diastolic diameter (LVEDD) with a positive correlation (rho 0.354, P<0.001). The IVCCI correlated with LMPI with a positive correlation (rho 0.230, P<0.001). The LMPI, RMPI, LVEDD and IVCCI did not correlate with the amount of IV fluids in the preceding 24 hours.

Enrolment lactate levels correlated positively with the total IV fluid volume received (rho: 0.323, P = 0.001) and also with enrolment ALT (rho: 0.764, P<0.001) and AST (rho: 0.773, P<0.001), but not with any of the cardio-haemodynamic parameters.

## Discussion

We have shown that myocardial impairment was not associated with recurrent shock but was associated with the secondary endpoint of respiratory distress after 3 days of the ICU admission. Lower stroke volume indices during the first 2 days of ICU admission and tachycardia were associated with both recurrent shock and respiratory distress. Higher lactate levels at ICU admission were also predictive for recurrent shock and respiratory distress. These results suggest patients with evidence of severe volume depletion at ICU admission including lower SVI, higher heart rates and venous lactates were more likely to develop recurrent shock and require more intravenous fluids–resulting in respiratory distress from a combination of plasma leakage and myocardial impairment, exacerbated by volume overload from fluid reabsorption in the recovery phase.

Cardiac functional assessment in patients with hypovolaemia is more challenging and hence our use of Doppler derived parameters, which have been shown to be less preload dependent [17]. Myocardial dysfunction was associated with respiratory distress but not with recurrent shock, suggesting the myocardial impairment was sufficient to play a role in fluid overload, following resuscitation and the associated respiratory compromise but not to contribute to the shock syndrome, which appears to be driven predominantly by intravascular volume depletion. These findings are comparable to other echo studies, including one study of Thai children which demonstrated 36% of patients with DSS had reduced systolic function and patients with cardiac impairment were more likely to have fluid overload [18]. The mechanisms underlying this transient myocardial dysfunction in dengue patients remain to be defined, but potential mechanisms may involve some or a combination of the following; myocardial depressant factors, myocardial interstitial oedema, abnormal coronary microcirculation and endothelial dysfunction and also abnormal calcium homeostasis [8, 19, 20].

Most patients admitted to ICU with severe dengue showed signs of intravascular volume depletion, as evidenced by low SVI, CI, and smaller LVEDD and higher IVCCI compared to follow-up. Ejection fractions however remained normal in all the groups, which may be explained by low end diastolic volume and/or diastolic dysfunction- both which may play a role in dengue. SVI was the most robust parameter associated with the severe outcomes of recurrent shock and respiratory distress. Heart rate was significantly higher at enrolment between patients with recurrent shock versus no shock. This confirms a previous study where higher heart rate was found to be useful in predicting children developing profound shock [21]. The IVCCI, although being higher in patients at enrolment compared to discharge, did not discriminate between shock and no shock and was not associated with clinical outcomes. IVCCI has also been shown to correlate with central venous pressure (CVP) and right atrial pressure (RAP) in children and adults [22, 23], and is useful in predicting fluid responsiveness, in mechanically ventilated patients and in spontaneously breathing patients [24, 25]. Due to the coagulopathy and thrombocytopenia in the majority of severe dengue patients, CVP carries a significant risk of bleeding and other non-invasive methods of assessing intravascular volume and guiding fluid therapy are urgently needed [26]. Portable bedside echocardiographic assessment of haemodynamics, particularly the SVI are useful in identifying patients with recurrent shock and could be considered as an alternative to invasive CVP monitoring.

We have shown venous lactates in dengue patients on the first day of admission to ICU is associated with severe outcomes of recurrent shock and respiratory distress. Lactate levels correlated with the total amount of IV fluids received, but did not correlate with other haemodynamic parameters.

The higher lactates likely represent severe volume depletion from plasma leakage causing tissue hypoperfusion, hypoxia and anaerobic glycolysis. In addition to hypoperfusion and excess production of lactate, another mechanism for hyperlactatemia in severe dengue may involve reduced hepatic clearance as moderate hepatic dysfunction occurs in severe dengue [27]. The liver may play a role in the hyperlactatemia in critical illness with circulatory failure, not only by reduced metabolism but also because the liver itself can produce lactate due to hepatic ischaemia. This is supported by our study which showed lactate levels correlated positively with both ALT and AST levels.

A study investigating patients with shock admitted to ICU, found higher lactate levels in patients with early hepatic dysfunction compared to those with no hepatic dysfunction, independent of haemodynamic severity parameters [28]. Altered microcirculation, which we have shown is worse in dengue patients with more severe plasma leakage [9], may play a role in the increased lactate levels, although further studies are required to link the microcirculatory perfusion abnormalities with higher lactate levels.

The current WHO guidelines for managing DSS recommend initial resuscitation with crystalloid fluids for compensated shock, followed by careful on-going assessment including serial HCT measurements and close monitoring of vital signs. Reassessing patients in shock and achieving predefined physiological targets has been a major focus of research in severe sepsis in the last 2 decades [29–31]. The ‘goals’ of resuscitation are currently being readdressed with emerging evidence that a conservative approach to fluid management has better outcomes in certain settings [32]. The balance of administering just sufficient intravenous fluid therapy to maintain haemodynamic stability while avoiding fluid overload and respiratory compromise is extremely difficult and additional cardiovascular monitoring using portable echocardiography in DSS would be beneficial. Stroke volume monitoring may provide improved targeted volume resuscitation. While serial HCT and vital sign monitoring are useful and widely applicable for resource constrained settings, intensive care facilities and associated technologies are improving in many dengue endemic areas, so additional non-invasive cardiovascular assessment is now possible and should be considered where available.

There were some limitations to our study. In order not to interfere with emergency management, some patients had the first echo study after initial fluid resuscitation had commenced. This may therefore underestimate some of the cardiovascular parameters. Secondly, due to restrictions on research blood sampling in paediatric patients, we were unable to take daily lactates so it was not possible to study lactate clearance times. In addition, due to the coagulopathy in many of severe dengue patients, we were unable to take arterial blood gases for assessment of metabolic acidosis to explore relationship with the high lactate levels. As the majority of patients enrolled in this study were children and young adults, the results may not be generalizable to older adult populations with dengue.

## Conclusion

In conclusion, this study has identified several simple non-invasive parameters that could assist risk prediction and help tailor management of dengue patients admitted to ICU. We have shown moderate cardiac dysfunction is common in ICU patients with dengue, particularly among adults. The cardiac dysfunction does not appear to play a major part in the haemodynamic instability of dengue shock but likely contributes to the development of fluid overload and respiratory compromise in some cases. Echo-derived volume assessment using stroke volume index combined with heart rate monitoring and venous lactate levels can help identify patients at high risk of recurrent shock. The clinical and therapeutic implications of these findings are potentially important, first as prognostic markers to guide fluid resuscitation and assist in the management of DSS as currently practiced, and second, these echo-derived haemodynamic endpoints could be used in future dengue fluid intervention trials designed to assess alternative strategies intended to improve DSS management and outcome.

## Supporting information

S1 Fig Study flow chart. (DOCX)

S1 Table Cardio-haemodynamic variables in DSS patients with and without respiratory distress over ICU admission. (DOCX)

S2 Table Daily Intravenous fluid volumes received by patients over the ICU admission. (DOCX)

S1 Appendix STROBE checklist. (DOC)

S2 Appendix Database. (XLS)
