# Supplementary material for: Cardio-haemodynamic assessment and venous lactate in severe dengue: Relationship with recurrent shock and respiratory distress
Source: PLoS Negl Trop Dis. 2017 Jul 10;11(7):e0005740. doi: 10.1371/journal.pntd.0005740 (PMC5519203; doi:10.1371/journal.pntd.0005740)
Supplement: S1 Fig — (DOCX) [file pntd.0005740.s001.docx]

Figure 1a Study Flow chart


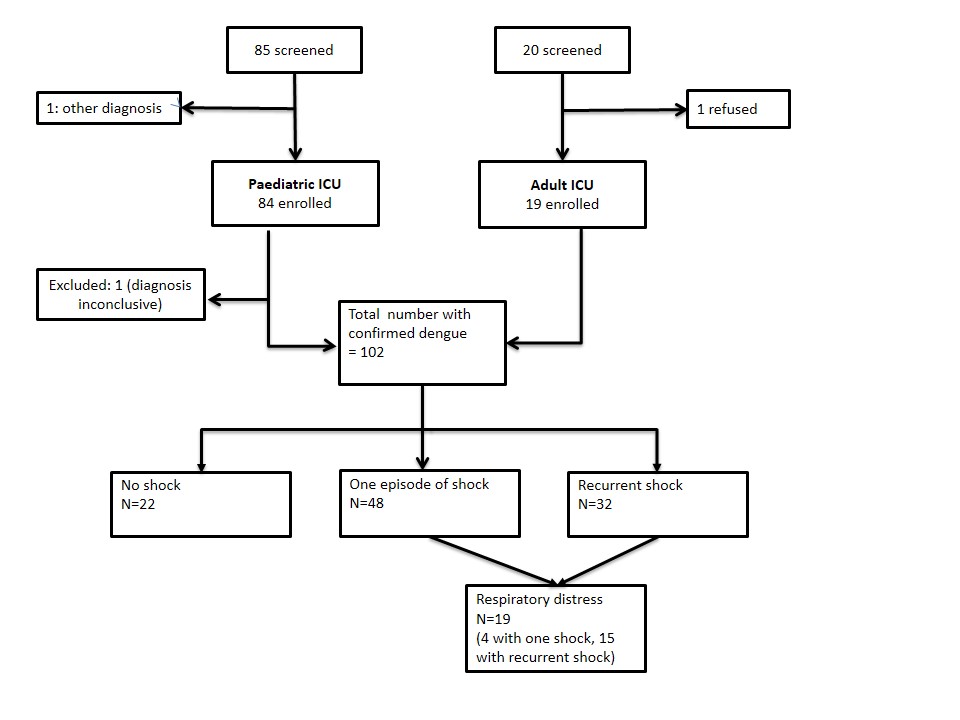


Study flow chart for patients enrolled in adult and paediatric intensive care unit (ICU)
