# Supplementary material for: Cardio-haemodynamic assessment and venous lactate in severe dengue: Relationship with recurrent shock and respiratory distress
Source: PLoS Negl Trop Dis. 2017 Jul 10;11(7):e0005740. doi: 10.1371/journal.pntd.0005740 (PMC5519203; doi:10.1371/journal.pntd.0005740)
Supplement: S1 Table — (DOCX) [file pntd.0005740.s002.docx]

Supplementary Table 1. Cardio-haemodynamic variables in DSS patients with and without respiratory distress over ICU admission

|  |  | **No Respiratory distress** |  | **Respiratory**  **distress** |  | |  | |  |  |
| --- | --- | --- | --- | --- | --- | --- | --- | --- | --- | --- |
|  | **n** | **(n=61)** | **n** | **(n=19)** | **Effect** | | **95%CI** | | **p** |  |
| **SVI ml/m^2^** |  |  |  |  |  | |  | |  |  |
| Day 1 | 56 | 22.9 (20.6, 25.6) | 12 | 22.3 (18.6, 24.4) | -0.87 | (-3.22, 1.48) | | 0.470 | | |
| Day 2 | 58 | 25.8 (22.9, 29.1) | 14 | 22.2 (20.5, 25.7) | -2.52 | | (-5.3, -0.25) | | 0.075 |  |
| Day 3 | 54 | 29.4 (25.2, 31.0) | 12 | 27.2 (25.0, 32.1) | -0.34 | | (-2.98, 2.31) | | 0.802 |  |
| Day 4 | 25 | 30.0 (27.7, 31.2) | 11 | 34.3 (32.1, 36.5) | 4.06 | | (1.25, 6.87) | | 0.005 |  |
| Day 5 | 8 | 31.3 (28.1, 34.2) | 6 | 36.0 (32.3, 38.1) | 3.84 | | (-0.69, 8.36) | | 0.096 |  |
| >Day 13 | 57 | 31.4 (29.8, 32.9) | 11 | 31.4 (30.5, 32.8) |  | |  | |  |  |
| **CI l/min/m^2^** |  |  |  |  |  | |  | |  |  |
| Day 1 | 56 | 2.1 (1.8, 2.4) | 12 | 2.4 (2.2, 2.5) | 0.27 | | (0.02, 0.52) | | 0.036 |  |
| Day 2 | 58 | 2.2 (1.9, 2.5) | 14 | 2.3 (2.1, 2.4) | 0.16 | | (-0.08, 0.41) | | 0.194 |  |
| Day 3 | 54 | 2.5 (2.2, 2.7) | 12 | 2.7 (2.3, 3.0) | 0.18 | | (-0.06, 0.43) | | 0.170 |  |
| Day 4 | 25 | 2.6 (2.2, 2.9) | 11 | 3.0 (2.9, 3.1) | 0.47 | | (0.07, 0.88) | | 0.023 |  |
| Day 5 | 8 | 2.4 (2.3, 2.8) | 6 | 3.0 (2.7, 3.2) | 0.15 | | (-0.31, 0.62) | | 0.516 |  |
| >Day13 | 57 | 2.8 (2.5, 2.9) | 11 | 2.7 (2.7, 2.9) |  | |  | |  |  |
| **LMPI** |  |  |  |  |  | |  | |  |  |
| Day 1 | 56 | 0.36 (0.26, 0.49) | 12 | 0.36 (0.30, 0.43) | -0.01 | | (-0.10, 0.07) | | 0. 748 |  |
| Day 2 | 47 | 0.36 (0.29, 0.476) | 10 | 0.48 (0.37, 0.55) | 0.07 | | (-0.02, 0.16) | | 0.140 |  |
| Day 3 | 44 | 0.29 (0.23, 0.35) | 12 | 0.48 (0.44, 0.50) | 0.21 | | (0.15, 0.27) | | <0.001 |  |
| Day 4 | 22 | 0.33 (0.26, 0.36) | 11 | 0.51 (0.50, 0.53) | 0.17 | | (0.12, 0.23) | | <0.001 |  |
| Day 5 | 8 | 0.26 (0.22, 0.32) | 6 | 0.34 (0.31, 0.36) | 0.09 | | (0.04, 0.14) | | 0.001 |  |
| >Day13 | 56 | 0.20 (0.16, 0.23) | 11 | 0.20 (0.18, 0.23) |  | |  | |  |  |
| **IVCCI** |  |  |  |  |  | |  | |  |  |
| Day 1 | 53 | 0.39 (0.34, 0.44) | 11 | 0.38 (0.33, 0.50) | 0.01 | | (-0.06, 0.08) | | 0.793 |  |
| Day 2 | 55 | 0.32 (0.28, 0.36) | 11 | 0.33 (0.28, 0.40) | 0.03 | | (-0.02, 0.09) | | 0.256 |  |
| Day 3 | 52 | 0.26 (0.21, 0.29) | 12 | 0.32 (0.26, 0.40) | 0.07 | | (0.03, 0.12) | | 0.003 |  |
| Day 4 | 26 | 0.24 (0.21, 0.27) | 11 | 0.23 (0.20, 0.26) | 0.01 | | (-0.03, 0.04) | | 0.735 |  |
| Day 5 | 8 | 0.22 (0.18, 0.23) | 6 | 0.22 (0.21, 0.24) | 0.02 | | (-0.03, 0.07) | | 0.427 |  |
| >Day13 | 57 | 0.22 (0.18, 0.24) | 11 | 0.20 (0.18, 0.22) |  | |  | |  |  |

*Data is presented as median (IQR). Each row corresponds to comparison of each variable by admission day based on linear regression, with adjustment for age, sex, illness day at enrolment. Effect (and 95%CI, p) corresponds to mean difference in variable between no respiratory distress and respiratory distress.*
