# Supplementary material for: Cardio-haemodynamic assessment and venous lactate in severe dengue: Relationship with recurrent shock and respiratory distress
Source: PLoS Negl Trop Dis. 2017 Jul 10;11(7):e0005740. doi: 10.1371/journal.pntd.0005740 (PMC5519203; doi:10.1371/journal.pntd.0005740)
Supplement: S2 Table — (DOCX) [file pntd.0005740.s003.docx]

**Supplementary Table 2. Daily Intravenous fluid volumes received by patients with and without shock and recurrent shock, over ICU admission**

|  |  | No shock  (n=22) |  | | Shock  (n=48) |  | Recurrent shock  (n=32) | | |
| --- | --- | --- | --- | --- | --- | --- | --- | --- | --- |
|  | n |  | n | |  | n |  | | |
| **Total IV per day** |  |  |  | |  |  |  | | |
| Day 1 | 4 | 710 (475, 1190) | 46 | | 2000 (1640, 2845) | 32 | 2825 (1322, 3965) | |  |
| Day 2 | 3 | 900 (555, 950) | 38 | | 1150 (585, 1908) | 32 | 2119 (1485, 3249) | | |
| Day 3 | 0 | - | 1 | | 500 | 7 | 1180 (970, 1285) | | |
| Day 4 | 0 | - | 0 | | - | 2 | 606 (533, 679) | | |
| Day 5 | 0 | - | 0 | - | | 2 | | 445 (262, 628) |  |

Data is presented as median (IQR) for continuous data. The results displayed are for new episodes of IV fluid started by day.
